# Interlimb Coordination during Forward and Backward Walking in Primary School-Aged Children

**DOI:** 10.1371/journal.pone.0062747

**Published:** 2013-04-23

**Authors:** Pieter Meyns, Kaat Desloovere, Guy Molenaers, Stephan P. Swinnen, Jacques Duysens

**Affiliations:** 1 Group Neuromotor Rehabilitation, Department of Rehabilitation Sciences, Faculty of Kinesiology and Rehabilitation Sciences, KU Leuven, Heverlee, Belgium; 2 Clinical Motion Analysis Laboratory, CERM, University Hospital Leuven, Leuven, Belgium; 3 Motor Control Laboratory, Movement Control and Neuroplasticity, Department of Kinesiology, Faculty of Kinesiology and Rehabilitation Sciences, KU Leuven, Heverlee, Belgium; 4 Department of Research, Development and Education, Sint Maartenskliniek, Nijmegen, The Netherlands; McMaster University, Canada

## Abstract

Previous studies comparing forward (FW) and backward (BW) walking suggested that the leg kinematics in BW were essentially those of FW in reverse. This led to the proposition that in adults the neural control of FW and BW originates from the same basic neural circuitry. One aspect that has not received much attention is to what extent development plays a role in the maturation of neural control of gait in different directions. BW has been examined either in adults or infants younger than one year. Therefore, we questioned which changes occur in the intermediate phases (i.e. in primary school-aged children). Furthermore, previous research focused on the lower limbs, thereby raising the question whether upper limb kinematics are also simply reversed from FW to BW. Therefore, in the current study the emphasis was put both on upper and lower limb movements, and the coordination between the limbs. Total body 3D gait analysis was performed in primary school-aged children (N = 24, aged five to twelve years) at a preferred walking speed to record angular displacements of upper arm, lower arm, upper leg, lower leg, and foot with respect to the vertical (i.e. elevation angle). Kinematics and interlimb coordination were compared between FW and BW. Additionally, elevation angle traces of BW were reversed in time (revBW) and correlated to FW traces. Results showed that upper and lower limb kinematics of FW correlated highly to revBW kinematics in children, which appears to be consistent with the proposal that control of FW and BW may be similar. In addition, age was found to mildly alter lower limb kinematic patterns. In contrast, interlimb coordination was similar across all children, but was different compared to adults, measured for comparison. It is concluded that development plays a role in the fine-tuning of neural control of FW and BW.

## Introduction

Forward (FW) and backward walking (BW) have been studied intensively in literature to shed light on the neural control of gait in different directions. These studies suggested that, in humans, the neural control of FW and BW may largely originate from the same basic neural circuitry [1], [2]. This suggestion was based on the finding that BW is basically FW in reverse [1], [3], [4]. The analysis of the kinematics of the leg movements have supported this idea, especially for the proximal joints [3]–[6]. This was achieved by comparing the movement traces of FW with those seen in time-reversed BW (revBW). Furthermore, even though electromyography (EMG) patterns of the separate leg muscles are not entirely similar between FW and BW, it was recently found that the five basic temporal EMG components, accounting for most variance of the total EMG wave form for FW and BW, correlated highly between the two walking directions [7]. One aspect that, to our knowledge, has not received attention is to what degree development plays a role in the maturation of the neural control of gait in different directions. It has been found that even infants younger than one years of age are able to walk in different directions when they were supported during walking [2]. Although previous literature suggested that the walking pattern in children is mature by the age of 2 or 3 [8], [9], recent evidence indicates that gait maturation (even in FW) occurs much later (by age 13) [10], [11]. Therefore, we questioned to what extent gait in different directions matures during childhood, when children are no longer supported during gait (i.e. in primary school-aged children).

Interest in the issue of gait maturation arose primarily from studies developing reference gait data sets to assess whether a child presents with normal gait characteristics for his/her age. Depending on the gait characteristics included in previous studies, maturation of gait has been suggested at different ages. For instance, in some studies reciprocal arm-swing was used as an indicator of gait maturity [8], [12], [13]. Infants at onset of independent walking are known to exhibit specific arm postures when walking [12], [14]. While gait matures, they initially fix their arms in a high guard position (external rotation at the shoulder, flexed elbows and hands at shoulder level) and gradually change to a low guard position (arms extended along the body without noticeable movement) [13]. At about 18 months most children adopt mature reciprocal arm swing movements during gait [8]. Similarly, the presence of heel-strike is a frequently used gait characteristic to assess gait maturity. The presence of a heel-strike is already evident in children 1.5 years of age [8], [15]. Gait characteristics such as cadence, step length and walking velocity, on the other hand, mature at a later age (4-7 years) [8], [15]. Lythgo et al. suggested that walking might even mature beyond the age of 13 based on their result that some gait characteristics such as stance duration, single and double support, were significantly different in 13 year old children compared to young adults [10]. Further support for the proposition that gait matures up to, and perhaps even beyond, 13 years of age is provided by the finding that 7 year-old children lack the neuromuscular maturity to produce an adult-like ankle peak plantar flexion moment and ankle power absorption/generation [16], [17]. Since these studies suggest that primary school-aged children might lack neuromuscular maturity when walking forward, we hypothesized that development might play a role in the maturation of BW as well.

Studies detailing the comparison of FW and BW focused mainly on the lower limbs. Recently, however, the arm movements during gait have been brought more into focus, since it has been proposed that arm swinging during gait originates from our ancestral quadrupedalism. Yet arm swinging is not vestigial because there is good evidence to support that arm swinging is important during walking since it was found to have a positive effect on energetic efficiency [18]–[20]. This occurs most probably by controlling total body angular momentum (i.e. to resist rotational torque about the body's vertical axis produced by the lower body) [21], [22]. Since arm swinging in humans is believed to result from locomotor networks (as in quadrupeds), this indicates that the neural control of arms is similar to that of the legs during gait [23]–[25]. Due to this similarity in neural control one might expect that the arm movements reverse in the same way as leg movements reverse when comparing FW with BW. From arm cycling studies it appears that this could well be the case [26] but, so far, for walking there have been no in depth studies. In walking, the limb loading conditions differ from those seen in cycling and therefore the question remains whether the results of cycling can be extrapolated. Hence, the present study is aimed at investigating the reversal of the kinematics of both the arm and leg movements during walking in two directions (FW and BW).

A first issue to be investigated in this respect is the coordination pattern. In adults walking at comfortable speeds the arm-to-leg swing ratio coordination is normally 1∶1 (i.e. one arm swing is associated with one leg swing) but at slow speeds this changes to 2∶1 (i.e. two arm cycles for one leg cycle) [27], [28]. In the current study we tested whether this 1∶1 arm-to-leg swing ratio during FW is preserved in children and in BW at a preferred walking speed. In addition the question arises whether the quadrupedal coordination pattern is maintained. In quadrupedal gait, the two most common coordination patterns are the trot and pace pattern. In the trot pattern the fore and hind limb on the same side of the body move in anti-phase (i.e. one moves forward while the other moves backward). In contrast, in the pace pattern, the fore and hind limb on the same side move in in-phase (both move simultaneous forward and backward) [29]. In healthy adults the arm swing obeys an anti-phase coordination with the leg on the same side in FW (i.e. like trot in quadrupeds) [30]. To our knowledge it has not been investigated whether the trot-like pattern is maintained in human BW and whether this pattern matures during development. In view of the evidence that human locomotion is basically organized as in animals such as cats and rats [31], it is important to consider this question in animal studies. This question of BW versus FW organization was investigated in several species but there is no consistent answer so far. For example, in cats walking backwards the trot pattern was preserved with a reversal of the order of paw contacts [32]. In contrast, mole rats have been shown to prefer the trot coordination mode in FW but they most frequently adopt the pace coordination mode in BW [33].

A related issue to be investigated is whether the kinematics of the arm swing during FW are consistent with a reversal of BW (as was shown for the legs). To investigate this, the traces of the arm in FW were correlated with those in revBW. In addition, a comparison was made of the peak amplitude, the mean position and the timing of the peaks of the traces of FW and BW. The hypothesis was that there would be a strong similarity between FW and revBW, consistent with the notion that FW and BW share common neural organizational features. We further hypothesize that the similarity in traces between FW and BW will gradually strengthen from pre-adolescence to adulthood.

In summary, the goal of this study was to determine to what extent development plays a role in the maturation of gait in different directions (FW and BW), with a special reference to both the lower and upper limb kinematics.

## Materials and Methods

Twenty-four children (12 males, 12 females; age 9.40 years ±2.16; weight 31.72 kg ±8.64; height 1.38 m ±0.14; mean ± standard deviation) and four adults (2 males, 2 females; age 29.86 years ±6.22; weight 68.85 kg ±6.21; height 1.74 m ±0.06) participated in this study. The inclusion of the adults was to verify whether the kinematics, as measured with our methods, corresponded to those already reported in the literature. Furthermore, this data were valuable as the protocol and methods used were the same for the adults as for the children.

All experiments were approved by the local ethical committee (“Commissie Medische Ethiek van de Universitaire Ziekenhuizen Leuven”) and were performed with the informed, written consent of the parents of the participants in accordance with the Declaration of Helsinki.

Participants were asked to walk at a preferred speed along a 10 meter walkway looking straight ahead either forward or backward. An eight camera Vicon system (Oxford Metrics, Oxford, UK) was used to measure three-dimensional full-body kinematic data (100 Hz). The total body “PlugInGait” marker set was used [34]. Three successful walking trials for each condition were used for further analysis. A successful trial included four consecutive foot strikes with full-marker-visibility, when the participant did not make excessive movements of the head, arms or trunk unrelated to walking. For both conditions the participant was granted some practice trials. For forward walking (FW) initial contact was taken as onset and end point of the gait cycle, while this was replaced by foot off for backward walking (BW).

### Data processing

The marker coordinates were filtered and smoothed using Woltring's quintic spline routine [35]. Workstation (Vicon Workstation 5.2 beta 20, Oxford Metrics, Oxford, UK) and Polygon software (Version 3.1, Oxford Metrics, Oxford, UK) were used to define the gait cycles and to determine the spatio-temporal parameters.

**Figure 1:**
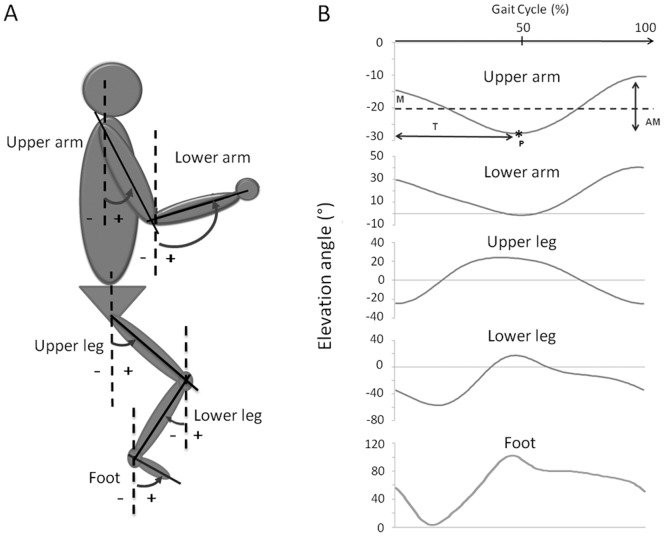
Representation of the measured elevation angles. Schematic presentation of the elevation angle i.e. the angle between the limb segment and the vertical (A), and an example of the time-courses of the elevation angle for the five segments on one side of the body (B). Note that all elevation angles have been measured for both sides of the body in all participants. The timing (T, horizontal double-headed arrow) of the first peak or valley (P, asterisk; depending on the segment studied), the amplitude (AM, vertical double-headed arrow), and mean (M, dashed line) of the overall trace were determined in all segments (represented here for the upper arm segment).

**Figure 2:**
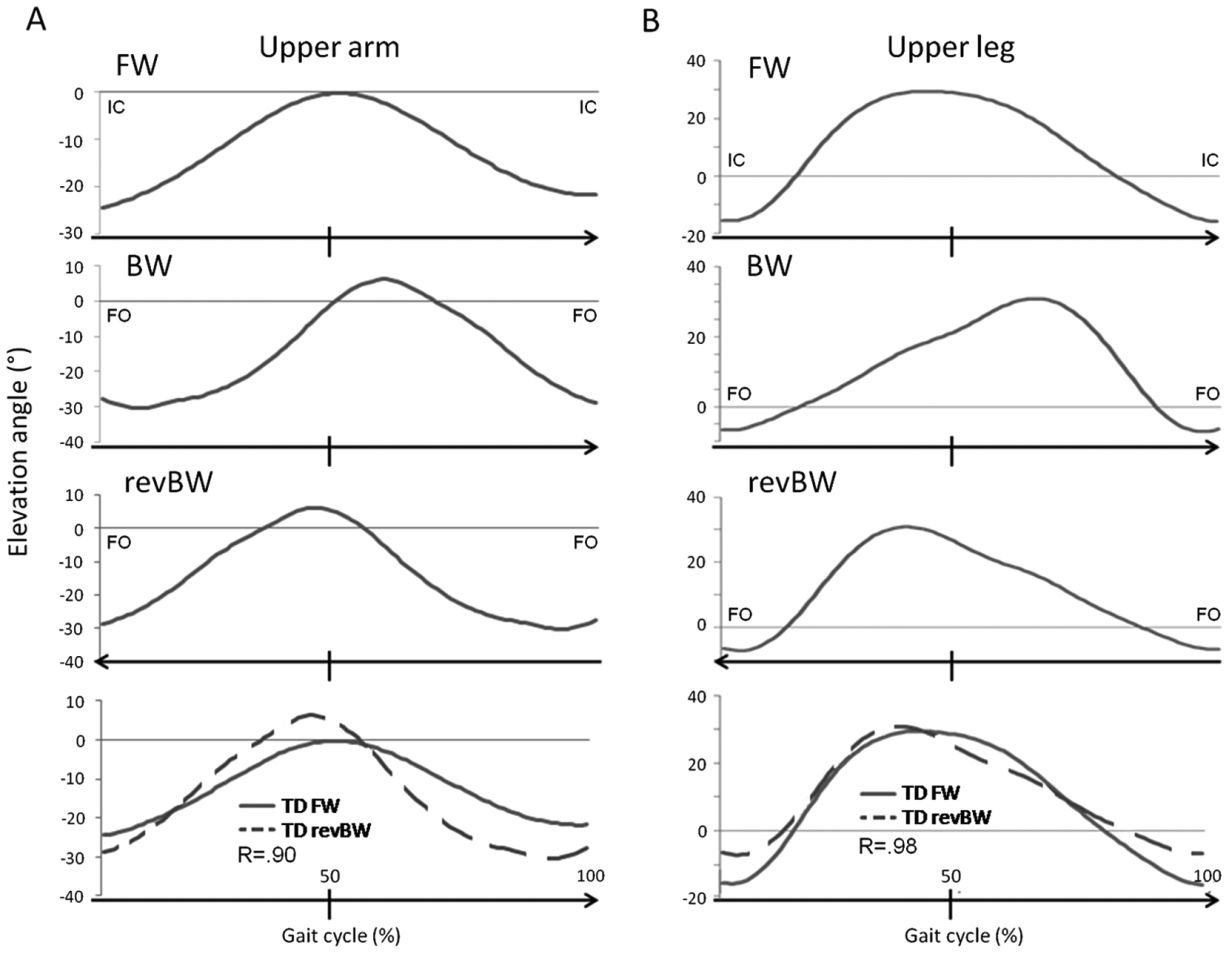
Example of the time reversal of an elevation angle trace for BW. The elevation angle trace for an upper arm (A) and an upper leg segment (B) were time-reversed for BW with respect to FW. There is a great similarity between the revBW and FW trace for the upper arm (represented by a high correlation coefficient R = 0.90), but the similarity for the upper leg is even higher (R = 0.98). Note that this time reversal was applied for all segments in all participants for BW. With FW = forward walking elevation angle trace, BW = backward walking elevation angle trace, revBW = time-reversed backward walking elevation angle trace, R =  Pearson correlation coefficient, IC = initial contact and FO = foot off.

The time-courses of the angular displacement of the upper arm (UA), lower arm (LA), upper leg (UL), lower leg (LL) and foot (FO) were recorded with respect to the vertical (i.e. elevation angles) in the sagittal plane (figure 1A & B). When the segment was rotated forward with respect to the vertical, this resulted in positive elevation angles (figure 1A). The elevation angle traces for BW were time normalized and reversed in time, so it was possible to directly compare the forward (FW) and reversed backward (revBW) traces (figure 2). In order to assess the similarity of these traces, for each participant the average revBW trace (of the three trials) was correlated to the average FW trace by means of Pearson correlation coefficients. Additionally, amplitude, mean and timing of the first peak or valley (depending on the segment studied) of the elevation angle traces were determined (figure 1B) and compared between the FW and revBW. The amplitude was defined as the difference between the maximum and minimum of the elevation angle trace, while the mean was specified as the average elevation angle over the gait cycle. The timing of the peak elevation was defined differently depending on the segment studied. For the dominant upper leg and lower leg, and for the non-dominant upper arm and lower arm the timing of maximal forward rotation with respect to the vertical (anteflexion) in the gait cycle was determined. On the other hand, for the non-dominant upper leg and lower leg, and for the dominant upper arm and lower arm the timing of maximal backward rotation with respect to the vertical (retroflexion) was determined. For both feet the percentage in the gait cycle of maximal plantar flexion was used.

In addition to angular displacement, angular velocity of the upper arm and upper leg segments with respect to the vertical (positive forward) were computed in the sagittal plane. All angular profiles were normalized in time as a percentage of the stride duration. Furthermore, they were normalized in amplitude (maximum and minimum 1 and -1, respectively). The phase angles of each segment were calculated from these normalized values. This enabled us to compute the continuous relative phase (CRP) between the different segments based on the technique as described by Stergiou [36]. The mean over the gait cycle (or the Mean Absolute Relative Phase [MARP]) was calculated from the CRP for trials where participants maintained a 1∶1 arm to leg swing ratio. This measure was used to analyze the timing of the interlimb movements. Coordinative stability was expressed by the standard deviation of the CRP (SDCRP) [37], [38]. Any trial with a different arm to leg swing ratio was excluded from further analysis. The data was analyzed in such a way that the patterns for both the dominant and the non-dominant limbs could be compared. The dominant side was defined as the side of the body where the arm was used to write or draw (right side for all children except one).

Since the position of the head can alter the movements and positions of both the arms and the legs, three parameters related to head rotation have been computed and compared between FW and BW. First, head rotation position over the gait cycle was determined whereby the value zero corresponds to having the head directed towards the line of progression, and positive and negative values correspond to head rotations to the non-dominant and dominant side, respectively. Second, variability of the head rotation position was defined as the standard deviation of the head rotation over the gait cycle. Third, maximal rotation to the non-dominant and dominant sides were computed. All variables were averaged over trials, for each participant at each walking condition.

### Statistical analysis

For the children and for the adults, paired t-tests were used to compare the walking speeds for the two walking conditions.

To evaluate the similarity of the elevation angle traces, the traces for time-reversed BW were correlated to the traces for FW using Pearson correlation coefficients. From these correlation coefficients, z-scores were calculated to determine possible outliers. One girl (11.5 years) presented with seven out of ten correlation coefficients that deviated two or more standard deviations from the mean and was, therefore, removed from all statistical comparisons. To compare each derived variable from the elevation angle traces (i.e. amplitude, mean, and timing of the first peak or valley) and the correlation coefficients of the two walking conditions we used a general linear model with two repeated measures factors (Walking Condition, Side of the Body). For the interlimb coordination measures (MARP and SDCRP), the same statistical model was used with only one repeated measures factor (Walking Condition). Since the walking speeds differed between the conditions, we included actual walking speed as a covariate in our analysis. The factor Age was also used as a covariate to assess its effect on the different measures for the two walking conditions. Tukey's post hoc comparisons were systematically applied. An α  = 0.05 was used to establish statistical significance.

## Results

### Walking speeds

When walking forward at their preferred speed, children showed a higher walking speed compared to walking backward (FW 1.19 m/s ±0.16 vs BW 0.82 m/s ±0.20, t(22) = −13.13, p<0.001). The adults did not walk significantly faster when going forward compared to going backwards (FW 1.16 m/s ±0.09 vs BW 1.08 m/s ±0.06, t(3) = 1.00, p = 0.39).

### Similarity between elevation angles traces for forward and time-reversed backward walking of arms and legs

**Figure 3:**
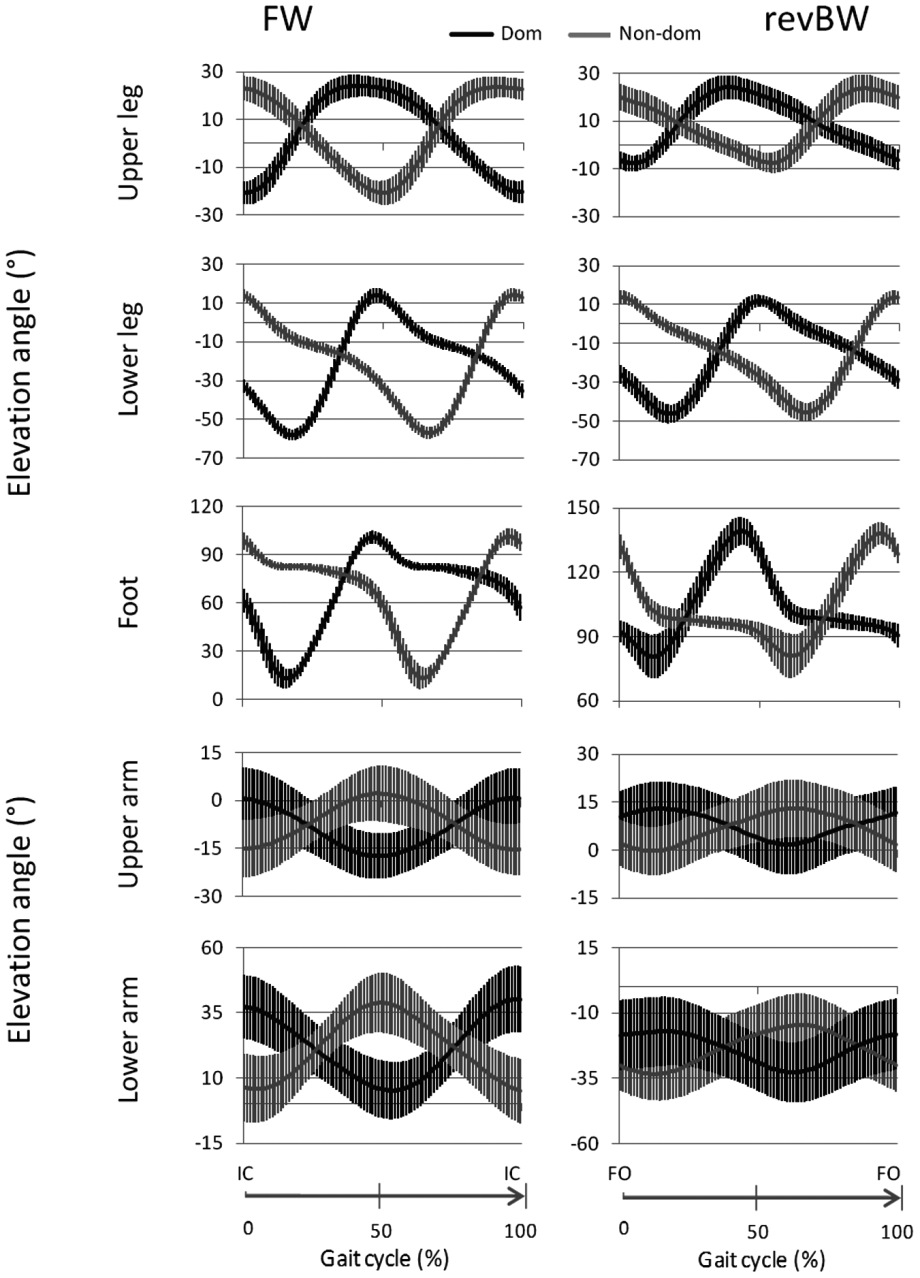
Group average of the upper and lower limb elevation angle traces. Group elevation angle traces (with positive and negative standard deviation) of the children for the upper and lower limb are presented on the dominant (black, Dom) and non-dominant (gray, Non-dom) side during FW (left column) and revBW (right column). With FW = forward walking elevation angle trace, revBW = time-reversed backward walking elevation angle trace, IC = initial contact and FO = foot off.

Group averages of the elevation angle traces of the children are presented in figure 3. Overall, the shape of the traces between FW and revBW were similar for all lower and upper limb segments.

**Figure 4:**
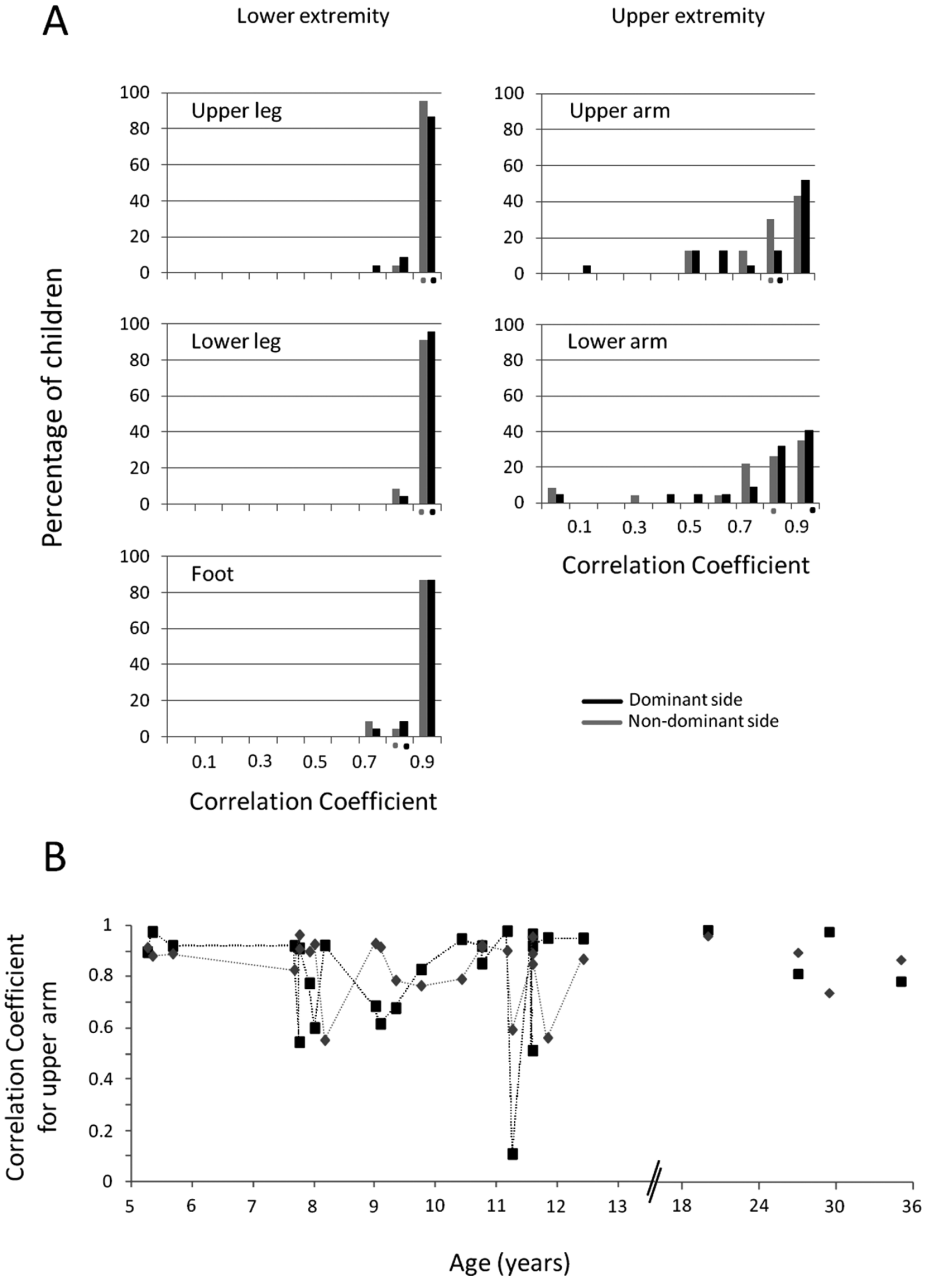
Percentage of children with their respective correlation coefficient between FW and revBW elevation angle traces. A. The percentage of children with their correlation coefficient are presented for the dominant (black, Dom) and non-dominant (gray, Non-dom) side, and for the upper (right column) and lower extremity (left column) segments. The average of results of the adults are presented by the black (dominant side) and gray (non-dominant side) dots underneath the x-axis of each graph. For representation purposes the correlation coefficients have been divided into ten subclasses (from 0 to 1, with steps of 0.1). Correlation coefficients were deemed very high when R>0.80, high when R = 0.60–0.80, medium when R = 0.40–0.60, weak when R = 0.20–0.40 and very weak when R<0.20. Note that most correlation coefficients are depicted on the right of the spectrum (even more for the lower extremity segments than for the upper extremity segments), which means that most children showed elevation angle traces for FW that correlated very well with revBW elevation angle traces. B. The respective correlation coefficient between the elevation angle trace of FW and revBW corresponding to each participant sorted by age for the upper arm. Note that no maturation effect is apparent during the development on the kinematic reversal from FW to BW.

Correlational analysis reveals that all children showed high to very high correlations coefficients between FW and revBW for the lower limb segments (i.e. upper leg, lower leg, and foot). The correlation coefficients ranged from 0.70 to 0.99 for all lower limb segments (figure 4A left), and were all highly significant (p<0.001). For the upper limb segments, most of the correlation coefficients were high to very high as well (i.e. 90.38% of the correlation coefficients for the upper arm, and 88.46% for the lower arm fell within the range of 0.6 and 1; figure 4A right). Nevertheless, for the upper extremity, some of the correlations were weak (1.92% for the lower arm segment with R = 0.20–0.40) or even very weak (1.92% for the upper arm and 5.77% for the lower arm with R<0.20).

The 25^th^ percentile of the values of the correlation coefficients was calculated for all segments. For the upper arm segments, 75% of the values of the correlation coefficients are above 0.68 (for dominant side) and 0.79 (for the non-dominant side). For the lower arm segments, 75% of the values of the correlation coefficients are above 0.76 (for dominant side) and 0.71 (for the non-dominant side). Finally, for the leg segments, the values of the correlation coefficients for the 25^th^ percentile ranged between 0.94 and 0.97.

Correlation coefficients were similar between primary-school aged children and adults (figure 4A). Age did not significantly affect the strength of the correlation coefficients between the elevation angle traces of FW and revBW for any of the segments (Upper arm: p = 0.75; Lower arm: p = 0.45; Upper leg: p = 0.57; Lower leg: p = 0.09; Foot: p = 0.06; see also figure 4B).

### Differences in elevation angle trace characteristics between forward and time-reversed backward walking

Comparison between FW and BW of the averages and standard deviations of the amplitude, mean and timing of the first peak (or valley) of the elevation angle traces for the different segments are presented in table 1.

**Table 1:** Comparison of elevation angle trace characteristics and interlimb coordination between FW and BW.

			FW	BW	F	p	p Age	p Age*Direction
Elevation angle trace	Upper leg	Amplitude (°)	45.78±4.20	32.54±5.25	9.33	***<0.01***	***0.04***	0.23
characteristics		Mean (°)	4.64±4.23	8.91±4.03	0.03	0.87	0.13	0.12
		Timing (%)	46.7±3.4	47.4±3.1	3.64	0.07	0.30	***0.03***
	Lower leg	Amplitude (°)	72.43±4.93	60.51±6.29	1.97	0.18	***0.002***	0.24
		Mean (°)	−20.07±2.08	−15.27±2.79	10.15	***<0.01***	0.69	0.51
		Timing (%)	57.5±1.3	59.0±2.5	2.99	0.10	***0.009***	0.64
	Foot	Amplitude (°)	89.18±7.68	60.07±10.71	5.13	***0.03***	***0.03***	0.12
		Mean (°)	66.59±3.06	76.17±3.93	5.57	***0.03***	0.70	0.93
		Timing (%)	40.7±1.5	42.3±2.5	0.79	0.38	0.15	0.79
	Upper arm	Amplitude (°)	18.72±9.84	14.42±7.38	0.10	0.75	0.37	0.95
		Mean (°)	−7.66±5.56	−7.34±5.64	0.43	0.52	0.73	0.60
		Timing (%)	48.6±4.7	47.5±7.1	0.01	0.95	0.07	0.62
	Lower arm	Amplitude (°)	34.94±15.73	20.41±10.86	0.01	0.93	0.30	0.45
		Mean (°)	22.22±4.03	23.78±7.37	1.35	0.26	0.99	0.99
		Timing (%)	52.3±6.0	49.4±9.4	0.17	0.69	0.60	0.40
Interlimb	Arms	Relative phase (°)	151.2±14.0	124.8±23.6	0.00	0.98	0.68	0.48
coordination		Coordinative stability (°)	18.8±10.6	29.8±8.7	0.04	0.84	0.39	0.70
	Legs	Relative phase (°)	141.6±4.4	132.3±8.1	0.00	0.99	0.54	0.24
		Coordinative stability (°)	20.0±2.8	27.8±4.3	0.18	0.68	0.54	0.59
	dom arm – dom leg	Relative phase (°)	150.8±10.4	136.4±16.0	1.07	0.31	0.81	0.93
		Coordinative stability (°)	19.2±6.3	28.6±8.6	0.09	0.77	0.42	0.51
	non-dom arm – non-dom leg	Relative phase (°)	145.1±13.5	140.0±12.4	4.29	0.05	0.42	0.93
		Coordinative stability (°)	22.1±5.8	24.5±6.9	6.49	***0.02***	0.65	0.62
	dom arm – non-dom leg	Relative phase (°)	33.1±10.2	48.7±13.8	2.74	0.11	0.28	0.65
		Coordinative stability (°)	22.5±5.7	28.8±7.7	5.48	***0.03***	0.54	0.98
	non-dom arm – dom leg	Relative phase (°)	33.5±13.5	47.4±11.3	0.66	0.43	0.44	0.43
		Coordinative stability (°)	20.9±6.5	29.5±8.8	0.11	0.74	0.34	0.68

Note that the elevation angle trace characteristics and interlimb coordination measures presented as follows: mean ± standard deviation. Abbreviations: FW  =  forward walking condition, BW  =  backward walking condition, F  =  value of the test statistic, p  =  calculated p-value for Direction (i.e. FW compared to BW), p Age  =  calculated p-value for the covariate Age, p Age*Direction  =  calculated p-value for the interaction effect between Age and Direction, dom  =  dominant, non-dom  =  non-dominant. Significant effects are presented in bold and italics.

For the lower extremity, the upper leg and foot segment showed significantly greater amplitudes of the elevation angle traces in FW compared to BW (see also figure 3), while no significant differences between amplitudes of the elevation angle traces were found for the lower leg segment. For the arms, the amplitude of the elevation angle traces of the upper extremity segments was similar for FW and BW.

To investigate whether there was an overall difference in positioning of the limb (such as a “guard position”) the mean over the gait cycle of the elevation angle traces were calculated (see also figure 3). No significant differences between FW and BW were found for the mean of the elevation angle traces in the upper leg segment. The mean of the elevation angle traces of the lower leg were rotated significantly more towards the posterior in FW than in BW. No significant differences in the mean of the elevation angle traces of the upper extremity segments were found between FW and BW.

To compare the timing of the most extreme positions, the time was calculated between the onset of the cycle and the maxima/minima of the elevation angle traces (see figure 1). As can be evaluated from figure 3, the timing of FW and revBW closely coincided. This was confirmed by the statistical analysis. There were no significant differences in the timing of the first peak (or valley) of the elevation angle traces (in percentage of the gait cycle) between FW and BW for any of the segments.

**Figure 5:**
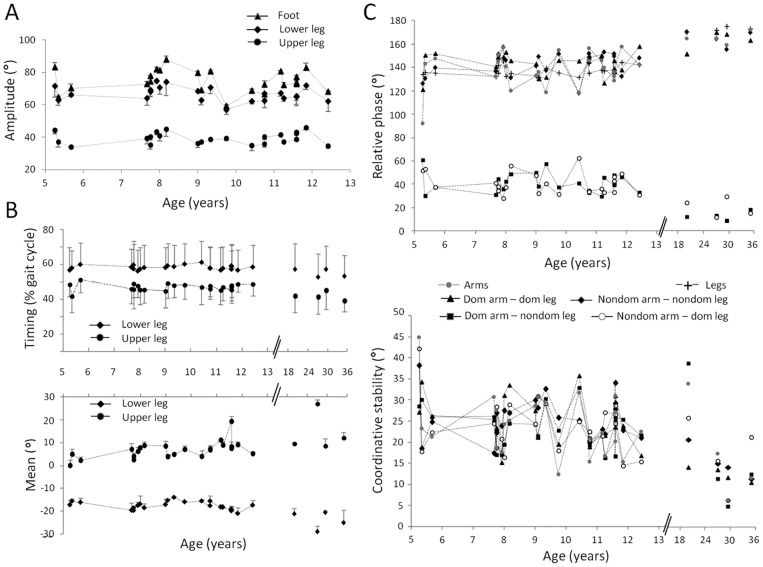
The effect of Age on elevation angle trace characteristics and interlimb coordination measures. A. The maximal elevation angle amplitude of the foot (Triangle), lower leg (Diamond), and upper leg (Circle) segment (averaged for Direction) decrease with age in children. B. When including the adults in the statistical group, several elevation angle trace characteristics showed a main effect of Age. B – upper graph. The timing of the first peak (or valley) of the elevation angle trace for the upper leg (Circle) and lower leg (Diamond) decreases with age. B – lower graph. The mean of the elevation angle trace for the upper leg (Circle) increases with age while the mean of the elevation angle of the lower leg (Diamond) segment decreased with age. C – upper graph. Additionally, when the adults were included, the coordination between all limb pairs improved with increasing age (arms [grey Circle], legs [+], Dom arm – dom leg [Triangle], Nondom arm – nondom leg [Diamond], Dom arm – nondom leg [square], Nondom arm – dom leg [empty Circle]). C – lower graph. Also, coordinative stability of all limb pairs (except for the legs) improved during aging. Note that the whiskers represent the standard deviation around the mean.

The covariate Age had only a limited effect on the characteristics of the elevation angle traces between FW and BW (see table 1 for p-values). Age had a significant effect on the amplitude of the upper leg, lower leg and foot segment (see figure 5A) and the timing of the lower leg elevation angle trace (figure 5B). In addition, a significant interaction effect of Age*Direction was found for timing of the upper leg elevation angle trace. Differences between children and adults (see figure 5B) were apparent for (1) the timing of the first peak (or valley) of the elevation angle trace of all segments (except the foot; main effect of age for Upper leg: p<0.001; Lower leg: p<0.001; Upper arm: p = 0.038; Lower arm: p = 0.002), and also for (2) the mean of all segments (except the upper arm; main effect of age for Upper leg: p = 0.002; Lower leg: p<0.001; Foot: p<0.001; Lower arm: p = 0.003), and for (3) the amplitude of the lower arm (p = 0.038) and lower leg segment (p<0.001).

### Interlimb coordination between arms and legs during FW and BW

Comparisons between FW and BW of the relative phase and coordinative stability of the interlimb coordination for the different limb pairs are presented in table 1.

One commonly used parameter to compare interlimb coordination is the frequency of arm swings during the step cycle or the arm-to-leg swing ratio [28]. Trials in which two arm movements occurred during one movement of the legs (i.e. with an arm-to-leg swing ratio of 2∶1) were identified. Six out of 72 trials showed a 2∶1 arm-to-leg swing ratio for FW, while for BW there were eight trials out of 72 with a 2∶1 arm-leg swing ratio. During FW, the double arm swing occurred on the non-dominant side in all 2∶1 arm-to-leg swing ratio walking trials, while during BW 2 of the 8 trials presented this phenomenon on the dominant side.

In a second analysis the relative phase was calculated. For this analysis the 2∶1 trials (i.e. 9.72%) were removed from further analysis. No differences in interlimb coordination were found between FW and BW for most limb pair combinations. There was one limb pair combination that tended to be different between FW and BW, i.e. the arm and leg on the non-dominant side. Tukey post-hoc test for this latter limb pair, however, did not reach significance (p = 0.13).

The coordinative stability was evaluated by calculating the standard deviation of the relative phase. For most limb pair combinations, no differences in coordinative stability were found between FW and BW. However, some limb pair combinations tended to differ for FW and BW with respect to coordinative stability. In particular, this was true for the arm and leg on the non-dominant side and the dominant arm combined with the non-dominant leg. Tukey post-hoc test for the arm and leg on the non-dominant side did not reach significance level (p = 0.16), while it did show a significant difference for dominant arm - the non-dominant leg limb pair (p = 0.002).

The covariate Age had no significant effect on either relative phase or coordinative stability (see table 1 for p-values). Differences between children and adults (see figure 5C) were clear for all limb pair combinations for the interlimb coordination measures (all: p≤0.001), and for all limb pair combinations (except the legs; p = 0.56) for coordinative stability (all: p≤0.01).

### Head rotation

When walking backwards some people might turn their heads to see where they are heading. Head rotation could influence the gait pattern and/or the arm movements. To rule out head rotation as a possible confounder, we checked whether there were differences between the two directions of walking.

Generally, children did not move their heads significantly more during BW compared to FW. Specifically, their head rotation position over the gait cycle and its variability were similar between conditions (head rotation position: FW −1.08°±4.63 vs BW -1.99°±5.34, F[1], [22] = 0.88, p = 0.36; head rotation variability: FW 1.72°±0.79 vs BW 2.08°±0.88, F[1], [22] = 2.51, p = 0.13), as was the maximal rotation towards the non-dominant and dominant side (maximal head rotation to non-dominant side: FW 2.78°±3.74 vs BW 2.77°±2.70, F[1], [22] = 0.0002, p = 0.99; to dominant side: FW 4.46°±3.46 vs BW 5.98°±5.24, F[1], [22] = 2.81, p = 0.11).

## Discussion

A first major result of the present study is that the interlimb coordination of FW is largely preserved in BW for both the primary school-aged children and adults. Both during FW and BW, only a minority of trials in children exhibited the 2∶1 arm-to-leg swing ratio. Inspection of these trials showed that in most cases the trial with this 2∶1 arm-to-leg swing ratio was the slowest of the three walking trials of the participant (nine out of fourteen). This indicates that in these few trials the participants might have walked at a slower walking speed than their actual preferred walking speed. In fact, overall walking speed in BW was slightly lower than in FW. Such a speed difference was not unexpected, since one of the important differences between FW and BW is the absence of vision of the surface where to step. Hallemans et al. (2010) showed that when sighted individuals were blindfolded (no vision condition) they showed slower walking speed [39]. This slower speed thus could explain why there was a small increase in 2∶1 arm-to-leg swing ratio coordination trials in the present study. However, for the main analysis of the data, the focus was on the 1∶1 arm-to-leg swing ratio coordination trials. Since, in these cases, the participants most likely did walk at a preferred speed, it is not expected that the (small) difference in walking speed between the conditions could have affected the results. Indeed, for the 1∶1 arm-to-leg swing ratio coordination trials, the data of BW were very similar to that of FW. During BW the arms swung reciprocally with each other and with respect to the leg on the same side, as is the case in FW. This pattern conforms to the quadrupedal trot pattern of interlimb coordination (arm and leg on the same side in anti-phase). The present data show that human children and adults do not switch coordination patterns when changing from FW to BW. This is in line with the view that human and animal locomotion (such as in cats and rats) are based on common principles possibly due to related ancestral neural networks [25], [31].

One exception with respect to the similarity between FW and BW related to the coordinative stability, as evaluated using the variability of the relative phasing between the limbs (i.e. standard deviation of the continuous relative phase). In BW, coordinative stability was significantly reduced for two out of six limb pair combinations as compared to FW. Basically, this also could have been due to the absence of vision of the path of the walkway in BW. Special attention was paid to assure that the children did not turn the head (to look at where they were going). Hence the present data were not contaminated by effects of head turns. However this means that the children did not have any vision of the surface they had to step on. Apparently, this did not affect the stability of the coordination of the legs but it appeared to have affected the interlimb coordination stability of the arms and legs. A study by Cockell et al. (1995) might elucidate this observation, since they indicated that the motor control system is organized in such a way that stability of the legs supersedes control of the arm movement patterns [40]. To summarize, the current results for most limb pairs did not differ between FW and BW with respect to coordinative stability. We only found a trend towards decreased coordinative stability for BW between the arm and leg on the non-dominant side, and a statistically significant difference in coordinative stability between the dominant arm and the non-dominant leg. Whether this statistical decrease is relevant remains an open question. It is concluded that even when walking direction is changed the coordination pattern is preserved in human walking. Some minor aberrations in the stability, however, can occur but they seem to be related to the absence of vision of foot positioning during walking.

A second major result concerns the question of reversed kinematics in BW. Indeed in the current study, we aimed to determine whether upper and lower limb kinematics during FW resemble those seen in time-reversed BW in children aged 5 to 12 years old and adults. We were the first to show that the upper limb kinematics during BW was organized as the time-reversed pattern during FW. Nevertheless, it is clear that the coupling was less tight for the arms than for the legs (resulting in lower correlations). Inspection of the data with the lower correlation coefficients revealed that in these cases usually a greater shift in phase was apparent between the FW and revBW trace. It is not unexpected that this shift in phase would occur somewhat more for the upper limbs, since they are not constrained by impact to the ground during swinging (contrary to the legs). Despite this shift in phase most children (at least 75%) showed a high to very high similarity in the kinematics between FW and revBW. In addition, the present analysis of the leg movements did show some differences for two features of the elevation angles traces of FW and revBW (i.e. mean and amplitude). Most differences were found in the foot segment. These differences do not necessarily confute the aforementioned hypothesis (i.e. that arm movements reverse in the same way as the leg movements do from FW to BW). They can, however, be explained by the modified control strategy at the ankle due to the altered foot roll off during BW compared to during FW. In BW there is an absence of the heel strike which strongly affects the role of specific muscles (e.g. tibialis anterior). Therefore, specific demands are imposed for the control of the ankle during BW [41]. In adults, knee and ankle joint angles have been found to be somewhat different in several other earlier studies [3]–[6]. Hence, the present data on primary school-aged children are in accordance to the results on adults and seem to be in line with the proposal of similarity similar neural control of locomotor leg and arm movements in children and adults.

### Maturation of FW & BW

The current results provide evidence that development plays a limited role in the maturation of gait in different directions in primary school-aged. All children showed the same global reversal of upper and lower limb kinematics even compared to adults when FW was compared with BW (as assessed with the correlational analyses). Age did show mild effects on a few specific kinematic gait characteristics measures in the group of primary school-aged children. This indicates that development had a maturation effect on these kinematic gait measures (i.e. amplitude of lower leg and foot elevation angles and timing of the first peak or valley of the upper leg and lower leg elevation angles). When the adults were taken into account, it appears that increasing age has an obvious effect on interlimb coordination and coordinative stability. The fact that this effect was only evident with the inclusion of the adults, together with the rather gradual slope of change from 5 to 12 years for most measures, indicates that neural control of walking is gradually fine-tuned throughout experiences in daily life towards an optimal coordination pattern, which might be related to optimal energy efficiency [42]. As Lythgo et al. previously suggested [10], gait may not be mature by the age of 13. Furthermore, based on the measures of interlimb coordination, it is possible that, in this view, gait may never be mature, since we believe the neural control to keep updating and fine-tuning throughout life.

### Neural mechanisms

Previous studies investigating FW and BW, tried to shed light on the mechanisms responsible for the neural control of gait. To date, there is debate about whether the lower legs during FW and BW are controlled by one functional network or by separate functional networks. Several studies that compare kinematics, kinetics and/or EMG in FW with time-reversed BW suggest that in humans basic neural control structures can simply reverse the automatism of FW to drive BW as well. Similarities in this reversal from FW to BW between humans and cats have prompted many researchers to believe that the rhythmic muscle activities during gait are generated by central pattern generators (“CPGs” for locomotion), i.e. specialized neural circuits located in the spinal cord [23], [43]–[45]. This CPG network controlling the limbs in rats and cats has been shown to be adaptive to changing directions of walking [46], [47]. In contrast, other researchers suggest that separate functional networks control forward and backward walking in humans. For instance, Choi and Bastian (2007) used a split-belt treadmill to adapt the right and left leg to FW and BW [48]. They found that the plasticity associated with locomotor adaptation is both leg and direction specific (i.e. walking adaptations are stored independently for each leg and do not transfer across directions), thus suggesting separate networks controlling FW and BW.

The present data do not allow identifying the mechanisms underlying the neural control of FW and BW, but focuses on the effect of development on the maturation of the neural control of walking in different directions. The current study shows that BW in the primary school-aged children and adults is performed mostly by reversing the patterns of movements of arm and legs as used in FW, but several gait characteristics are gradually adapted, most likely depending on varying biomechanical and neuromuscular constraints during aging.

Some limitations should be considered when interpreting the current results. In the current experimental setup, electromyography of the arm muscles was not measured. Therefore, we were unable to determine whether the kinematic similarities of arm swinging during FW and BW are due to the muscles being driven in reverse order or due to the mere biomechanical interactions between the legs and arms. Nevertheless the present data indicate indirectly that active control of swing was present in these children. Indeed, in most cases a 1∶1 arm-to-leg swing ratio was maintained and from previous work on adults it is known that this type of coordination is related to active muscle contribution, whereas the 2∶1 coordination mode (which occurs at slow walking speeds) relies on a passive mechanism [28]. This result has been confirmed by Kuhtz-Buschbeck et al., who indicated that there is an important active component to the swinging of the arms during walking. They have based their conclusion on the fact that electromyography signals persisted when the arms were immobilized during walking [49].

Another limitation concerns walking speed. Walking speed in BW was not similar to FW because preferred walking speed was imposed on the currently evaluated participants. Therefore, all gait cycles were time-normalized and walking speed was implemented as a covariate into the statistical model. Children performed practice trials of BW and FW until they felt confident. The difference in coordinative stability between FW and BW, might have been caused by the relative lack of experience with BW. Further research is required to investigate whether an extensive training period would improve coordinative stability.

Finally, it should be recalled that our kinematic analysis had its limitations. Elevation angles and interlimb coordination were determined from angular displacements of segments in the sagittal plane only. This was based on the finding that the largest arm movements were present in the anterior–posterior direction in the currently evaluated children [50]. Therefore, we opted for a simplified kinematics approach that was deemed satisfactory for kinematic analysis in typically developing children. In addition such analysis can be performed in children with cerebral palsy [38], [50], [51], thereby allowing the investigation of the question whether damage to the corticospinal pathways interferes with the reversal as described here. This will be the topic of a forthcoming study.

## Conclusions

The current results support the notion that, already from an early age on (>5 years), BW is organized as the reverse of FW, both for arm and for leg movements. Development plays an important role in the fine-tuning of the neural control of walking in different directions.
